# Islam’s weight in global history: A response to Sidaway

**DOI:** 10.1177/20438206231177082

**Published:** 2023-05-23

**Authors:** Pol Llopart i Olivella, Till Mostowlansky

**Affiliations:** 30525 Graduate Institute of International and Development Studies, Switzerland; 30525 Graduate Institute of International and Development Studies, Switzerland

**Keywords:** Anthropology, decolonial, geography, global history, Islam

## Abstract

In this commentary, we discuss three major themes that Sidaway raises in his article, ‘Beyond the Decolonial: Critical Muslim Geographies’: the problem of Muslims as ‘others’; the fraught role of religion as a universal category; and Muslim geographies as perceived in area studies and global history. Along these lines, we argue that Sidaway makes a number of important interventions aimed at changing the social science focus on Muslims in the West, highlighting the importance of Islamic concepts, and dislocating spaces of Islam from predefined geographical areas. After a critical discussion of the specific approaches presented in the article, we follow up on Sidaway's encouragement to think beyond the decolonial. We see this as an invitation to formulate our own vision of a new global history of Islam that takes into account traces of the influence of Muslims and of Islam more broadly speaking from Indigenous Australia to China to the Americas, and from everyday culture in Europe to extinct empires in Iberia, Sicily, and the Balkans. From this perspective, we argue, a more serious engagement with the multitude of global Islamic influences beyond Muslim communities might turn into a powerful force of decolonization.

James Sidaway's (2023) article on Muslim geographies constitutes an important contribution to the debate on decoloniality in geography. His proposal to scrutinize erasures and exclusions, and in turn to highlight Islamic concepts, spaces, and historical connections, is timely. It also adds a novel angle to the decolonial debate, which Sidaway views as rooted in literature on Latin America. Going beyond this existing framework, Sidaway grounds his argument in interdisciplinary approaches ranging from the anthropology of Islam to Black geographies. In this commentary, we discuss these approaches from our perspective as social anthropologists working on transregional Muslim connections that emerge from practices of charity, aid, and solidarity. We center our comments on three major topics that Sidaway raises at different points in the article: the problem of Muslims as ‘others’; the fraught role of religion as a universal category; and Muslim geographies as perceived in area studies and global history.

An important part of Sidaway's article consists of a critical assessment of the existing social science literature on diasporic Muslims in the West (e.g. Rana, 2018). He emphasizes that many such publications are informed by migration and diaspora studies, and foreground *difference* (i.e. Muslims’ perpetual ‘otherness’ vis-à-vis the Western spaces that they inhabit). This strand of research tends to highlight Muslims as a minority set against the backdrop of a supposed non-Muslim norm. We agree with Sidaway that, in this epistemological framework, engagement with Islamic scholars and knowledge production has been peripheral.

As Sidaway further points out, the way religion features in this literature has often been schematic. He argues that, in this regard, Islam has been inserted into a comparative approach to religion that ‘imposes a Eurocentric frame…that is then projected onto a range of categories and communities as though they are counterparts or analytically interchangeable’. While Sidaway is referring specifically to the role of religion in geography, the same could also be said about many other disciplines in the social sciences that have grappled with a perceived ‘return of religion’ to politics and public spaces in the 2000s (Ebaugh, 2002). Sidaway's critique of an – often implicit – secularist approach to religion is correct insofar as the distinction between what is religious and what is not religious is already the outcome of the historical process of secularizing knowledge (Asad, 2003; Agrama, 2012). The social sciences have struggled with this conundrum precisely because their emergence within the academy is inextricably linked to the rise of a secularist frame (Asad, 1993; Tayob, 2018).

Looking for a decolonial way to overcome this frame, Sidaway argues that instead of reducing Muslim geographies to mere religious geographies, we should turn to Islamic concepts. He identifies *dīn* as one such central concept, referring to Al-Attas's (2020) definition of *dīn* as a ‘coherent system (of action and knowledge) and judicious power, inclination, habit, disposition, as well as obligation – which includes a related set of commercial forms associated with mercantile capital’ (Sidaway, 2023). On the one hand, this approach is commendable insofar as it highlights the diversity of possible ways of thinking about Islam. It thereby importantly advocates for serious engagement with Islamic scholars not only as subjects of analysis, but also as theorists in their own right, on a par with social scientists. On the other hand, invoking *dīn* without historicizing its changing, time-bound interpretations runs the risk of once again essentializing and exoticizing Islam. This comes at the cost of downplaying Islam's internal diversity and Islamic scholars’ longstanding, creative, and productive engagement with religion and other universalist categories (Asad, 1993, 2018).

This trade in concepts – Islamic and otherwise – has always been part and parcel of particular geographies that challenge the regional containers of area studies. In his article, Sidaway discusses, among other conceptual frameworks, the ‘Balkans-to-Bengal complex’, a term Shahab Ahmed (2016: 81) proposes to amalgamate a multiplicity of societal forms connected by a common paradigm of Islamic life and thought. Sidaway turns a hopeful eye to the Balkans-to-Bengal framework as an example of an approach based on a geographical and cultural area and understood under the rubric of ‘intellectual histories shaping Islam's conceptions of itself’. His intention is to offer pathways for reconfiguring Muslim geographies beyond imperial and colonial gazes. However, this attempt to open up a new horizon of Muslim presence across diverse geographical regions also overlooks longstanding and expansive concepts such as diaspora, roots/routes, and movement, concepts that are employed in the Black decolonial writings with which Sidaway aims to engage (Allen and Jobson, 2016).

While he identifies lacunae comprising excluded geographical and intellectual histories – for instance, those of the Sahel and sub-Saharan Africa – Sidaway overlooks the fact that Ahmed's (2016: 83) examination is primarily concerned with a particular ‘case study’ in a specific space and time and at a certain historical ‘stage’. Moreover, other authors since Ahmed have sought to destabilize and decompartmentalize colonial boundaries and histories. Expanding on Ahmed, they have explored vast networks of interconnectivity amidst political fragmentation (Kia, 2020; Ziad, 2021). Their proposals have sought to look at and trace movements that go beyond the well-established regional areas, such as the Oxus-to-Cairo region (Hodgson, 1974), which Sidaway also references, or ‘West Asia’ (Marsden and Mostowlansky, 2019), revealing ‘an interconnected web of social-intellectual-religious movements’ (Ziad, 2021: 16). Furthermore, Ahmed (2016: 541) himself had already encouraged the reader to move forward and ‘transport the theoretical and conceptual lens that we have fashioned in the Balkans-to-Bengal, so as to bring it to bear upon other societies of Muslims in other times and places’. Thus, while the Balkans-to-Bengal complex is an appealing inspiration, reifying the concept into a regional field petrifies otherwise flexible and fluid spaces of interconnectivity in which one could explore a plethora of movements, intellectual exchanges, and political histories. Following Ahmed, we regard this and related concepts as exploratory and generative tools that serve to unveil emergent networks rooted in and moving through particular times and spaces.

In his article, Sidaway also proposes to look beyond Asian geographies and toward Africa. He endorses Ware's (2014) call to take West Africa seriously as a site of Islamic authority and knowledge. Sidaway recognizes the importance of African participation in the intellectual history of Islam and, in doing so, he seeks to destabilize the notion of ‘African Islam’ as marked by exceptionalism and peripherality. He thereby also contests academic uses (and abuses) of syncretism (i.e. the process of merging different traditions) as a defining feature of African Islam. To abandon particularistic notions and situate African intellectual production within the wider history of Islam, he reminds us that it is not only African Islam, but ‘all formations of Islam [that] are in some mode or other syncretic’. While we understand his proposal to extend the descriptor ‘syncretic’ to practices in all Islamic traditions and spaces, we argue that it risks falling back into the binary of local versus orthodox Islam. In other words, we recognize the merits of reflecting on the role of syncretism as a sometimes underacknowledged ‘popular strand of Islamic thought and life’ (Taneja, 2018: 8). However, we would also like to point out that the concept of ‘syncretism’ is a potentially misleading way of framing Islamic lives (McIntosh, 2009). The existence of syncretism can only be sustained by its opposite, that is, by the presupposition of an authentic and coherent tradition immutable through space and time. From this perspective, syncretism appears incomplete, incoherent, and inauthentic. This view corresponds to Jefferson-Tatum's (2020: 507) argument that syncretism as an analytical notion constructs local traditions as a problematic *other* and feeds on a ‘modern materialization and transfiguration of older imperialist projects of comparison and classification’ that replicates colonial discourses.

Accordingly, the move toward decoloniality should not bolster problematic concepts such as syncretism; it should, rather, aim to criticize, historicize, and denaturalize them. Moreover, postcolonial and decolonial theories have argued that hybrid forms of knowledge are generative and bear the potential to displace Western rationality from its position as the sole framework of social existence and thought (Grosfoguel, 2006). In finding alternatives to syncretism and similar binaries, we turn again to Ahmed (2016), who has pointed out that the study of Islam is disproportionately focused on prescriptive authority, constrained by legalist understandings, and marked by the overuse of binaries. This prescriptive fixation on law derives from the emergence of the modern nation-state – both secular and religious – as a ‘*definitive and constitutive authority* [that] is necessarily vested *in legal discourse* – every law becomes an act of defining and constituting Islam, the state, *and, thereby, the Muslim citizen*’ (Ahmed, 2016: 530, emphasis in original). In contrast, Ahmed (2016: 282) proposes the notion of ‘explorative authority’ to account for the contradictions, the multiplicity of truths and values, as well as the hybrid and counter-hegemonic self-expressions and ethics within Islam (see also Taneja, 2018: 6; Jankowsky, 2021: 201).

Our criticism notwithstanding, we find ourselves in agreement with many points raised in Sidaway's article. Perhaps most importantly, we are fully aligned with the larger, urgent endeavor of balancing concepts emerging from debates in the social sciences with those theorized and practised in Muslim settings. In the last paragraph, Sidaway argues that ‘the historical and geographical richness of Islam points the way to something even more expansive than the decolonial’. He observes a subversion of previous spatial and civilizational categories, and sees in the ‘richness of Islam’ the potential of invoking ‘something better, involving deeper scrutiny of circulations and intersections that crisscross humanity’. How exactly Islam fosters subversion and how this should result in ‘something better’ – perhaps a transformed discipline of human geography? – remains unsaid. Sidaway thus leaves it to his readers to come up with their own pathways that transcend the decolonial. Reflecting on this, we feel that going beyond existing decolonial discourse in this particular case would necessarily entail an acknowledgement of Islam's weight in global history. Wherever we look in the past and present, from Indigenous Australia to China to the Americas, from everyday culture in Europe to extinct empires in Iberia, Sicily, and the Balkans, Muslims and Islamic influences have played a prominent role. Islam's traces are to be found in all these spaces in, among other manifestations, material culture, legal history, and mapping. A more serious engagement with the multitude of global Islamic influences beyond Muslim communities could be a powerful force of decolonization.
